# Chronic Kidney Disease in Common Variable Immunodeficiency: a Multicenter Study

**DOI:** 10.1007/s10875-025-01890-2

**Published:** 2025-05-23

**Authors:** Chiara De Renzis, Renato Finco Gambier, Antonietta Gigante, Carla Maria Deiana, Gianluca Lagnese, Lorenzo Gatti, Giulia Garzi, Giulia Costanzo, Chiara Pagnozzi, Stefania Nicola, Luisa Brussino, Giuseppe Spadaro, Marcello Rattazzi, Davide Firinu, Francesco Cinetto, Cinzia Milito

**Affiliations:** 1 Department of Translational and Precision Medicine, “Sapienza” University of Rome, Rome, Italy; 2 Rare Disease Referral Centre, Internal Medicine I, Department of Medicine, Cà Foncello Hospital, @AULSS2 Marca Trevigiana, DIMED - University of Padua, Treviso, Italy; 3 Department of Medical Science and Public Health, University of Cagliari, Monserrato, Italy; 4 Department of Translational Medical Sciences, University of Naples Federico II, Naples, Italy; 5 Department of Molecular Medicine, “Sapienza” University of Rome, Rome, Italy; 6 Department of Medical Sciences, University of Torino & Mauriziano Hospital, Torino, Italy

**Keywords:** Common Variable Immunodeficiency, Chronic Kidney Disease, Dysregulation, Inflammation, Aging

## Abstract

**Purpose:**

There are few reports of renal involvement in Common Variable Immunodeficiencies (CVID) and, when present, is due to infections, inflammation, or treatments. The aim of this study was evaluating the prevalence of chronic kidney disease (CKD) and to identify CVID-related clinical, laboratory and therapeutic features inducing it.

**Methods:**

A multicenter observational retrospective study on 367 adult CVID patients from five Italian Referral Centers for Primary Immunodeficiency.

**Results:**

CKD was identified in 23 (6.27%) patients that were older (*p* < 0.001), had arterial hypertension (*p* < 0.001), diabetes (*p* = 0.002), dyslipidemia (*p* = 0.002), presented different ultrasound abnormalities (*p* < 0.001) and received predominantly intravenous immunoglobulins (IVIG) (*p* = 0.016). Regarding CVID infectious and non-infectious manifestations, CKD patients presented a higher frequency of COPD (*p* = 0.008). In the CKD group, the median absolute count of total lymphocytes (*p* = 0.015), the percentage of total B (*p* = 0.028) and transitional B cells (*p* = 0.008) were lower. By binomial logistic regression analysis adjusted for age, CKD patients tend to develop autoimmune cytopenia, had lower B cells percentage, increased Neutrophil-to-lymphocyte ratio and received more frequently trimethoprim-sulfamethoxazole antibiotic prophylaxis. By multivariate analysis, only autoimmune cytopenia was independently associated with CKD.

**Conclusion:**

The prevalence of CKD in CVID is due to aging, age-related comorbidities, disease-related immune dysregulation and inflammation. Our results suggest evaluating renal function in all CVID patients, and mostly in those with a higher “inflammatory” burden.

**Supplementary Information:**

The online version contains supplementary material available at 10.1007/s10875-025-01890-2.

## Introduction

Common Variable Immunodeficiency (CVID) is the most common primary symptomatic antibody deficiency in adulthood. It is characterized by hypogammaglobulinemia, poor response to pathogens and vaccinations and by a heterogeneous spectrum of clinical manifestations including recurrent infections, malignancies and immune dysregulation such as autoimmune and inflammatory disorders and granulomatous manifestations [1, 2].

There are few reports of renal involvement in CVID [3–5]. The prevalence of renal diseases in Primary Antibody Deficiencies (PADs) accounts for about 2%, including granulomatous disease, immune complex or membranoproliferative glomerulonephritis, nephrotic syndrome, end-stage renal disease and amyloidosis [6–8].

Renal biopsy is rarely performed in CVID patients, but when performed, the predominant pathologic findings include membranous glomerulopathy and tubulointerstitial nephritis.

The pathogenesis of renal injury is still unclear [9]. Different factors have been evoked: alteration of permeability causing disruption in glomerular podocyte leading to nephrotic syndrome, increased chronic inflammation, T cell infiltration with the development of autoimmunity probably related to a failure in the microbial antigen elimination and production of high levels of tumor necrosis factor and interferon gamma [10].

In a recent study by Materne et al., renal disease was reported in 6.8% of PAD patients; in particular: 35.7% had chronic kidney disease (CKD), 32.9% nephrolithiasis, 12.9% nephritis and 35.7% other renal complications. Patients with CKD had lower counts of total lymphocytes, CD3 + and CD4 + T cells, CD19+, CD20 + and CD27 + IgD- B-cells whereas patients with nephritis had lower counts of absolute lymphocytes, CD19 + B cells and IgE levels. CKD and nephritis were associated with an increased prevalence of non-infectious complications including autoimmune, gastrointestinal and pulmonary disease and in conclusion, CKD was associated with reduced numbers of class-switched memory B cells and a more severe immunophenotype [11].

Intravenous (IVIG) or subcutaneous (SCIG) replacement treatment represents the mainstay therapy in CVID patients. IVIG are useful in reducing infections but may induce renal damage, especially in older patients with preexisting kidney disease [12].

Moreover, renal damage might be also related to the presence of excipients in IVIG preparations including sucrose, albumin, glycine, maltose and glucose added for preventing aggregation and dimer formation. In particular, glucose was associated with acute kidney injury (AKI) whereas, maltose, even if well tolerated, may induce osmotic nephropathy and AKI [13, 14]. Neutrophil-to-lymphocyte ratio and platelet-to-lymphocyte ratio are novel, cost-effective, easily obtainable, and widely available markers of inflammation [15]. These inflammatory biomarkers are useful in monitoring CKD and have been associated with CKD progression towards end-stage renal disease and with higher mortality rate [16, 17].

Based on this background, we designed an observational study to describe the prevalence of CKD in a multicenter cohort of adult CVID patients. In addition to risk factors leading to renal injury in the general population, we analyzed whether CVID-related clinical, laboratory and therapeutic features might influence it.

## Methods

We conducted a multicenter observational retrospective study on a cohort of 367 caucasian adult patients with a diagnosis of CVID regularly followed in five Italian Referral Centers for Primary Immunodeficiency (AOU Policlinico Umberto I in Rome, Ospedale Ca’ Foncello in Treviso, Policlinico Universitario of AOU di Cagliari, AOU Federico II in Naples, AO Ordine Mauriziano in Turin). CVID diagnosis was established according to ESID criteria [18]. The observational period for the prospective phase was 01 June, 2022–01 December, 2023.

We collected data on age, gender, time of CVID diagnosis, CVID related phenotypes according to Chapel’s classification [19], presence of hypertension and blood pressure values (arterial hypertension was defined in accordance with the 2023 ESH/ESC guidance) [20], diabetes, episodes of AKI, Body Mass Index (BMI, calculated from weight (kg) and height (m) and expressed in kg/m^2^), recurrent urinary tract infections (≥ 2 episodes in six months or ≥ 3 in one year), Ig replacement therapy, and use of concomitant treatments, including antibiotic prophylaxis. The antibiotic prophylaxis was prescribed in a subgroup of patients of our centers according to literature suggestions [21]. Trimethoprim-sulfamethoxazole was preferred in patients with lower lymphocyte count, particularly if requiring treatment of immune-mediated complications.

We evaluated the following laboratory parameters: hemoglobin, platelet count, leukocyte count (including neutrophils and lymphocytes), lymphocyte subsets according to EUROclass classification [22], creatinine, and urine tests.

Renal function was defined by estimated glomerular filtration rate (eGFR), considering serum creatinine (Scr) values. To estimate GFR, we used the new Chronic Kidney Disease Epidemiology Collaboration equation [23].

Neutrophil-to-lymphocyte ratio and platelet-to-lymphocytes ratio were calculated at the time of eGFR estimate.

The presence and stage of CKD were characterized according to the Kidney Disease Outcomes Quality Initiatives guidelines as follows: stage 1 (> 90 ml/min), stage 2 (60–89 ml/min), stage 3A (45–59 ml/min), stage 3B (30– 44 ml/min), stage 4 (15–29 ml/min), and stage 5 (< 15 ml/min). According to guidelines, CKD is defined as a decreased GFR of less than 60 mL/min/1.73 m^2^ for at least 3 months [24].

During the study time, patients underwent at least one renal ultrasound in which we evaluated changes in cortico-medullary differentiation and longitudinal bipolar diameters, signatures of kidney damage. In addition, we focused on other abnormalities including mild pyelectasis, nephrolithiasis, and cysts. Renal ultrasound together with liver and spleen ultrasound are part of routine care for CVID patients and are performed periodically.

Genetic analysis were performed by next-generation sequencing carried out on an Ion Gene Studio S5 system (Ion Torrent, Thermo Fisher Scientific, Waltham, Mass) using a custom Ion AmpliSeq On-Demand panel (Thermo Fisher Scientific), designed to detect single nucleotide changes and small insertion-deletion variants in genes associated with antibody deficiency and common variable immunodeficiency.

The study project was approved by the Local Ethics Committee. All patients signed the informed consent. The study was conducted in accordance with the Declaration of Helsinki and the last version of Good Clinical Practice.

### Statistical Analysis

Patient characteristics were summarized using medians, standard deviations, interquartile ranges (IQR), and percentages as appropriate. Chi-squared tests of independence or Fisher’s exact tests were used for categorical data as appropriate. Mann-Whitney U was used for unpaired continuous data. Binomial logistic regression models were fitted to calculate odds ratios (OR) with 95% confidence intervals (95%CI). Multivariable logistic regression analysis was then performed, to confirm the findings after adjustment for age. Statistical significance was considered as a p value < 0.05. The Shapiro-Wilk test was used to ass the normality of the analyzed variables.

## Results

### Patients’ Characteristics

We enrolled a total of 367 CVID patients regularly followed at 5 Referral Centers (Rome, Treviso, Cagliari, Naples and Turin). Our cohort was composed of 162 (44%) male and 205 (56%) female with a median age of 51 years (IQR 38–62).

In our cohort, the median creatinine concentration was 0.78 (IQR 0.69–0.91) mg/dl with a median eGFR value of 98 (IQR 84–109) mL/min. We classified patients according to eGFR; a total of 23 (6%) patients presented CKD: 15 (4%) patients were CKD stage 3a, 6 (1.5%) were CKD stage 3b, and 2 (0.5%) were CKD stage 4 or higher.

A total of 7 patients (2%) presented changes in cortico-medullary differentiation and longitudinal bipolar diameters evaluated at renal ultrasound, whereas 84 patients (23%) presented other renal ultrasound abnormalities including mild pyelectasis, nephrolithiasis, and cysts.

Of note, we observed a total of 8 (2%) cases of AKI who recovered. In particular, in 3 out of 8 patients, AKI was due to drugs including mesalazine for inflammatory bowel disease (IBD) and meropenem for pneumonia.

Regarding comorbidities, we identified 40 (11%) patients who suffered from recurrent urinary infections, 88 (24%) with arterial hypertension, 24 (6.5%) affected by diabetes, 149 (41%) overweight and obese patients, and 95 (26%) with dyslipidemia.

All data about laboratory results and associated comorbidities are provided in Table 1 and Supplementary Table 1.

**Table 1:** Demographic characteristics, comorbidities, immunological phenotyping, infectious related clinical data, and data on Immunoglobulin replacement therapy, compared between CVID patients with or without CKD. Mann-Whitney U test was used for continuous variables, Chi squared for categorical variables.

	Total cohort (*n* = 367)	No CKD (*n* = 344)	CKD (*n* = 23)	*p* value	OR (IC95%)
**Demographic characteristics**					
Age, median (IQR)	51 (38–62)	51 (38–61)	75 (62–77)	**< 0.001**	**1.11 (1.07–1.17)**
Female sex, n (%)	205 (56%)	191 (56%)	14 (61%)	0.617	1.25 (0.52–2.96)
Overweight, n (%)	149 (41%)	141 (41%)	8 (35%)	0.557	0.77 (0.32–1.86)
**Non-immunological comorbidities**					
Hypertension, n (%)	88 (24%)	76 (22%)	12 (52%)	**< 0.001**	**3.83 (1.63–9.03)**
Diabetes, n (%)	24 (6%)	19 (6%)	5 (22%)	**0.002**	**4.74 (1.59–14.1)**
Dyslipidemia, n (%)	95 (26%)	83 (24%)	12 (52%)	**0.002**	**3.64 (1.52–8.74)**
Cases of AKI, n (%)	8 (2%)	3 (1%)	5 (22%)	**< 0.001**	**31.4 (6.95–142)**
**Immunological phenotyping**					
Diagnostic delay (years), median (IQR)	6 (1–16)	7 (1–16)	8 (4–13)	0.836	1.00 (0.99–1.01)
CVID duration (years), median (IQR)	10 (5–18)	10 (5–18)	11 (6–24)	0.232	1.03 (0.99–1.07)
IgG at diagnosis (mg/dl), median (IQR)	300 (167–424)	300 (167–434)	270 (224–319)	0.290	0.99 (0.99-1.00)
IgA at diagnosis (mg/dl), median (IQR)	9 (2.5–25)	9 (2–25)	12 (5–23)	0.992	1.01 (1.00-1.03)
IgM at diagnosis (mg/dl), median (IQR)	21 (10–37)	22 (9–37)	17.5 (12–32)	0.914	1.00 (0.99-1.00)
Complicated phenotype, n (%)	178 (48.5%)	164 (48%)	14 (61%)	0.225	1.70 (0.72–4.03)
Splenomegaly, n (%)	171 (47%)	159 (46%)	12 (52%)	0.511	1.34 (0.56–3.18)
AI cytopenia, n (%)	106 (29%)	95 (28%)	11 (48%)	0.053	2.28 (0.97–5.34)
Enteropathy, n (%)	80 (22%)	78 (23%)	2 (9%)	0.124	0.31 (0.71–1.35)
AI systemic disease, n (%)	24 (6.5%)	22 (6%)	2 (9%)	0.656	1.39 (0.30–6.32)
ILD, n (%)	60 (16%)	54 (16%)	6 (26%)	0.202	1.87 (0.71–4.96)
**Infectious manifestations**					
COPD, n (%)	67 (18%)	58 (17%)	9 (39%)	**0.008**	**3.11 (1.29–7.54)**
Bronchiectasis, n (%)	124 (34%)	115 (33%)	9 (39%)	0.603	1.26 (0.53–2.99)
Recurrent UTI, n (%)	40 (11%)	37 (11%)	3 (13%)	0.729	1.24 (0.35–4.36)
Antibiotic prophylaxis, n (%)	67 (18%)	60 (17%)	7 (30%)	0.108	2.11 (0.83–5.36)
Trimethoprim-sulfamethoxazole, n (%)	16 of 66 (24%)	13 (4%)	3 (13%)	0.115	3.28 (0.79–13.7)
Azithromycin, n (%)	50 of 66 (76%)	47 (13%)	3 (13%)	0.753	0.70 (0.18–2.69)
**Immunoglobulin replacement therapy**					
Ig RT, n (%)	352 (96%)	329 (96%)	23 (100%)		
SCIG, n (%)	242 (69%)	231 (67%)	11 (48%)	0.523	0.754 (0.32–1.80)
IVIG, n (%)	110 of 355 (31%)	98 (28%)	12 (52%)	**0.016**	**2.74 (1.17–6.41)**
Maltose-stabilized product, n (%)	40 of 110 (36%)	36 (10%)	4 (17%)	1.000	0.91 (0.26–3.17)
IgG trough level (mg/dl), median (IQR)	731 (645–882)	735 (650–878)	710 (592–918)	0.585	1.00 (0.99-1.00)
IgRT mg/kg/month, median (IQR)	370 (300–428)	370 (300–426)	340 (300–470)	0.775	1.00 (0.99-1.00=
**Ultrasound renal evaluation**					
Changes in cortico-medullary differentiation and longitudinal bipolar diameters at US, n (%)	7 (2%)	3 (1%)	4 (17%)	**< 0.001**	**24.7 (5.12–119)**
Any other US abnormalites*, n (%)	84 (23%)	74 (22%)	10 (43%)	**0.015**	**2.81 (1.18–6.66)**

* mild pyelectasis, nephrolithiasis, cysts. Statistically significant results in bold

The median age at CVID diagnosis of the whole cohort was 39 years (IQR 28–50 yrs) with a median disease duration of 10 years (IQR 5–18 yrs). According to the revised Chapel et al. classification, 189 patients (51.5%) presented the “infection only” phenotype, whereas 178 (48.5%) had a complicated phenotype including autoimmune cytopenia, enteropathy, polyclonal lymphoproliferation. In addition, to further explore potential differences between these two phenotype groups we compared the laboratory tests that are presented in Supplementary Table 2.

Regarding CVID related-manifestations, 165 (45%) patients presented chronic lung disease, in detail, 124 (34%) exhibited bronchiectasis, 59 (16%) had a history of interstitial lung disease (ILD) and 67 (18%) were diagnosed with chronic obstructive pulmonary disease (COPD). Moreover, a history of autoimmune cytopenia was present in 106 (29%) patients. All immunological comorbidities are displayed in Table 2.

**Table 2:** Laboratory data including B cells sub-populations identified according to EUROclass study [22] in the study population compared between CVID patients with or without CKD. Mann-Whitney U test was used for continuous variables, Chi squared for categorical variables.

	Total cohort (*n*=)	No CKD (*n* = 344)	CKD (*n* = 23)	*p* value	OR (IC95%)
Hb (g/dl), median (IQR)	13.8 (12.6–14.9)	13.8 (12.6–14.9)	13.5 (12-14.4)	0.146	0.81 (0.64–1.02)
Creatinine, median (IQR) mg/dl	0.78 (0.69–0.91)	0.77 (0.68–0.88)	1.21 (1.06–1.38)	**< 0.001**	**1.04 (0.92–1.18)**
eGFR (mL/min/1.73m2), median (IQR)	98 (84–109)	99 (87–110)	50 (41–55)	**< 0.001**	**0.81 (0.74–0.88)**
Platelet-to-lymphocyte ratio, median (IQR)	119 (86.2–159)	119 (86.5–156)	121 (83.1–211)	0.481	1.00 (0.99-1.00)
Neutrophil-to-lymphocyte ratio, median (IQR)	2.06 (1.5–3.08)	2.05 (1.5–2.94)	3.2 (1.43–4.87)	0.183	1.14 (1.01–1.28)
ALC (cells/mm^3^), median (IQR)	1530 (1090–2052)	1544 (1100–2052)	1044 (609–1475)	**0.015**	**1.00 (0.99-1.00)**
T cells %, median (IQR)	77.5 (70-82.3)	77 (70–82)	81 (75.3–86.5)	0.105	1.01 (0.99–1.04)
CD4 + T cells %, median (IQR)	41 (32–49)	41 (32–49)	39 (29.8–50.8)	0.653	0.98 (0.94–1.03)
CD8 + T cells %, median (IQR)	30 (24-38.8)	30 (24–38)	34 (23.8–52)	0.289	1.04 (0.99–1.09)
B cells %, median (IQR)	9 (4–13)	9 (5–13)	4 (4-7.8)	**0.028**	**0.89 (0.80-1.00)**
Sm B cells %, median (IQR)	2 (0.9–4.9)	2 (0.9-5)	1.5 (0.8–3.5)	0.579	0.97 (0.82–1.15)
Mz B cells %, median (IQR)	7 (2–15)	8 (2–19)	5.5 (2–18)	0.633	0.98 (0.94–1.04)
CD21low B cells %, median (IQR)	6 (3.9–12)	6 (3–12)	6 (2–16)	0.906	1.01 (0.97–1.07)
Naive B Cells %, median (IQR)	66 (33–84)	66 (33–85)	64 (46–81)	0.793	1.00 (0.98–1.03)
Tr B cells %, median (IQR)	1 (0.2–2.5)	1.2 (0.3–2.6)	0.05 (0-0.12)	**0.008**	**0.002 (5.45e-7-7.08)**
Sm B < 2%, n (%)	118 (32%)	112 (33%)	6 (26%)	0.520	0.73 (0.28–1.90)
B < 1%, n (%)	24 (7%)	22 (6%)	2 (9%)	0.655	1.39 (0.31–6.33)

B cells sub-populations were identified according to EUROclass study: Sm B cells: Switched memory IgD − IgM − CD27+; CD21low B cells: Activated CD21lowCD38low; MZ B cell: Marginal zone IgD + IgM + CD27+; Tr B cells: Transitional CD38 + + IgMhigh; Naive B cells: Naïve IgD + IgM + CD27− [22]. Statistically significant results in bold.

**Figure caption list**

In the 28% (103/367) of the enrolled population a screening for monogenic forms of CVID was performed. Results were available for 83/367 patients (23%): 7/23 (30%) CKD patients and 76/344 (22%) patients without CKD. The most frequent mutation identified was a TACI variant, found in 22/76 (29%) non-CKD patients and in 3/7 CKD patients (43%), without a statistically significant difference between the 2 groups (*p* = 0.425). Of the total TACI variants, 23 were monoallelic mutations and 2 were biallelic mutations.

Moreover, in the CKD group (but also in a patient without CKD) a VUS on Casp 10 gene was found. No other genetic mutations were found in the CKD group.

A total of 352/367 (96%) patients were on IgG replacement treatment (IgRT); in detail, 242 out of 355 (68%) received subcutaneous immunoglobulins (SCIG or fSCIG) and 110 out of 355 (31%) were on IVIG treatment. Among patients treated with IVIG, 40 (36%) received a maltose-stabilized product. The median IgG trough level of the whole cohort was 731 mg/dl [IQR 645–882 mg/dl] with a median IgRT monthly dosage of 370 mg/Kg (IQR 300–428 mg/Kg), without any significant difference between the two routes of administration. A total of 67 (18%) patients were on antibiotic prophylaxis with azithromycin or trimethoprim-sulfamethoxazole. The remaining immunological characteristics of our cohort are shown in Table 2.

### Comparison of patients’ variables in relation to CKD

We then compared the demographics and clinical features of patients with and without CKD (Table 1).

We observed that patients with CKD were significantly older (75 vs. 51, *p* < 0.001). Considering the significant differences in the median age between patients with and without CKD, we decided to perform a binomial logistic regression analysis, adjusted for age (Supplementary Table 3).

**Fig. 1:**
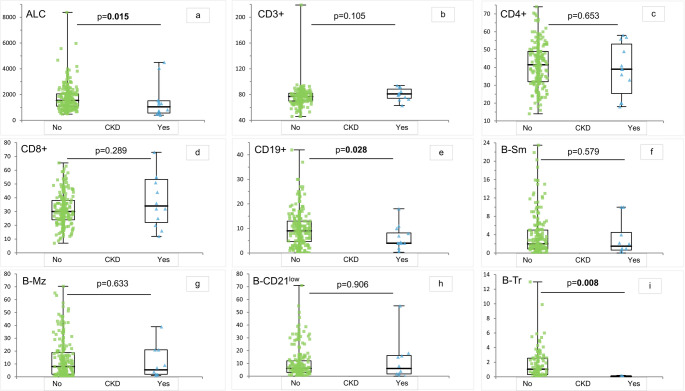
T e B cell immunophenotype in patients with and without CKD

We observed that patients with CKD were significantly more prone to develop AI cytopenia (*p* = 0.008, OR 3.54 (1.38–9.08)). Moreover, we confirmed that CKD patients had lower B cells percentage (Fig. 1) and more frequently received trimethoprim-sulfamethoxazole antibiotic prophylaxis. In addition, age-adjusted analysis highlighted that CKD group had an increased neutrophil-to-lymphocyte ratio. Finally, as expected, patients with CKD had a significantly higher prevalence of changes in cortico-medullary differentiation and longitudinal bipolar diameters at ultrasound.

All other variables that were significantly different in the CKD group, compared to no CKD (Table 1), lost their significance after adjustment for age. These included cardiovascular and metabolic co-morbidities such as arterial hypertension, diabetes, and dyslipidemia. Further variables showing significant differences between CKD and no CKD group include changes in cortico-medullary differentiation and longitudinal bipolar diameters and other US abnormalities at US, immunologic parameters as median absolute count of total lymphocytes and percentage of the transitional B cell (Fig. 1) and IVIG administration as replacement therapy. Among IVIG products, the use of maltose-stabilized product was not different between the two groups (Table 1).

Finally, we performed a multivariate analysis including all the significant variables associated with CKD after correction for age, apart from specific cortico-medullary differentiation and longitudinal bipolar diameters US abnormalities. This analysis showed that only a history of autoimmune cytopenia was independently associated with the presence of CKD (Supplementary Table 4).

In addition, analyzing the effect of CVID-infection-related comorbidities on the enrolled cohort, we observed a significant association between COPD and CKD (*p* = 0.008). All results are displayed in Table 1.

## Discussion

Chronic kidney disease is defined as abnormalities of kidney structure or function, present for a minimum of 3 months, with implications for health. The best-recognized causes include diabetes, hypertension, and aging. Decreased renal function is represented by GFR < 60 ml/min per 1.73 m^2^ (GFR categories G3a–G5) with ultrasound changes (renal length and parenchymal thickness reduction) [23].

Global prevalence of CKD in the general population was estimated at 9.1% (8.5 − 9.8%), in 2017, by a systematic analysis [25, 26].

In Italy, the prevalence of CKD is around 7.5% of the adult male population and 6.5% of the adult female population that accounts today for about 2.5 million people affected [27].

According to the Global Burden of Disease study, the prevalence of CKD will increase, over time, driven by population aging, diabetes and hypertension. For this reason, the number of people developing kidney failure and requiring kidney replacement therapy is estimated to increase to more than 5 million by 2030 [25, 26].

Prior studies of CKD in primary antibody deficiencies are scarce and limited to small cohorts. In particular, CKD was reported in a small percentage of patients and in about 2% of CVID patients enrolled in a cohort of 240 UK patients [3–5, 28].

In our cohort of 367 CVID patients, we observed a CKD (GFR < 60 ml/min) prevalence of 6.3%.

Our results are mostly in line with data from the general Italian population and with those recently published [11] on the USIDNET cohort of PAD patients, where 6.8% of patients had renal complications. However, Materne et al. considered in their study all PADs, investigated the prevalence of renal disease with a more general definition, including not just CKD but also nephrolithiasis, nephritis, and other renal diseases and did not specify a GFR-based criterion for CKD. Thus, our CKD prevalence, evaluated only in CVID, has to be considered as higher [11].

In our cohort, CKD is almost completely attributable to age, even when other risk factors such as hypertension, diabetes and dyslipidemia also present in the general population were present.

The association between renal disease and these comorbidities is in line with the progressive aging of our cohort, a probable consequence of improved survival observed in the last years due to the efficacy of modern tailored care and of treatment availability [29]. This is likely the main reason why our prevalence of CKD shows a trend towards the global prevalence and seems to move away from the prevalence reported in older studies.

Due to the retrospective design of the study and to the lack of precise information in the clinical records, particularly before CVID diagnosis and regarding patients with a long disease history, we were not able to weigh the number and severity of infections in relation to CKD development. We could only compare the well-known markers of infectious risk such as IgG and IgA levels at diagnosis, memory B cells, IgGTL, bronchiectasis and need for antibiotic prophylaxis. These parameters did not differ significantly between CKD and no CKD patients. Analyzing non-infectious CVID-related complications, we did not observe a significant association between complicated phenotype and CKD, but, also after age-adjustment, we found an increased risk for patients with autoimmune cytopenia that, compared to the other immune-mediated complications, almost always requires glucocorticoids and eventually immune-suppressive treatment. In addition, lymphocytes subsets, the median absolute count of total lymphocytes and the percentage of total B and transitional B cells were significantly reduced in the CKD group whereas class-switched memory B cells were lower, even if without statistical significance. In the general population, CKD has been associated with a reduced total lymphocytes count [30]. Class-switched memory B cells reduction is known to be associated with a higher risk of developing immune dysregulation, including autoimmune cytopenia [11, 31]. A reduction of transitional B cells, as found in our CKD group, seems also to be associated with immune dysregulation [32]. These cells are indeed involved in regulatory processes including the conversion to regulatory T cells. We could speculate that the alteration of the immunological phenotype of a subgroup of CVID patients may influence the inflammatory background and favor chronic organ damage, as already highlighted in pulmonary, gastroenteric tract and cardio-vascular manifestations [3, 33–34]. The lymphopenia may also explain the higher rate of trimethoprim-sulfamethoxazole prophylaxis use in CKD patients. Accordingly, we also reported a higher prevalence of COPD and CKD in our cohort, a well-known association also in the general population [35]. In fact, the vicious circle infection-inflammation [36], typical of COPD, might explain the use of trimethoprim-sulfamethoxazole antibiotic prophylaxis, in the attempt to prevent lung damage and remodeling [37]. Notably, compared to azithromycin, trimethoprim-sulfamethoxazole prophylaxis was commonly used also to prevent PCJ infection in patients with severe lymphopenia or in those receiving glucocorticoids or immune-suppressive treatment (for example, in case of AI cytopenia). We know that inflammation is a common feature of CVID, explained both by immune dysregulation and infectious events [38]. In line with an increased inflammatory milieu and with the presence of lymphopenia, the neutrophil-to-lymphocyte ratio, a prognostic marker of worse renal outcomes [18], and CKD showed a positive correlation by binomial logistic regression analysis, corrected for age, supporting a more pronounced inflammatory state in patients with renal and cardiovascular comorbidities [33, 39].

Regarding Ig replacement therapy, as supported by the age-adjusted analysis, the higher prevalence of IVIG use in the CKD cohort might be mainly related to the older age of the CKD patients, who tend to prefer a hospital-based rather than a home-based immunoglobulin administration. In fact, in Italy, IVIG is administered only in a hospital setting, allowing for a more frequent contact between patients and medical staff. This is supported also by other studies where older patients, displaying a severe phenotype and a high rate of complications, still preferred the IVIG compared to the SCIG treatment [40].

Finally, in CVID patients who developed CKD, as expected, we observed a significant association with ultrasonography alterations including changes in cortico-medullary differentiation and longitudinal bipolar diameter at US, mainly related to arterial hypertension and cardiovascular risk factors, as well as pyelectasis and nephrolithiasis. This latter might induce an obstruction of the urinary tract that alters the urinary flow and might further promote infections and renal damage over time in patients with increased infectious burden [41].

Our study has some limitations, mainly due to the retrospective design and unavailability of data exactly reporting the time of CKD onset and its evolution, as well as the exact number of infectious episodes and antibiotic courses. Furthermore, results of the genetic screening were not available for the whole cohort. However, the number of enrolled patients, coming from a single Country and with homogenous ethnicity, makes it a valuable source of information on a poorly investigated issue.

In summary, CKD in CVID is largely related to aging and age-related comorbidities, together with disease-related immune dysregulation and inflammation. So, autoimmune cytopenia, lower B cell percentage, increased neutrophil-to-lymphocyte ratio and need of trimethoprim-sulfamethoxazole rather than azithromycin antibiotic prophylaxis may help to identify a subgroup of patients with a more “inflammatory” phenotype that over time promote the development and worsening of CKD, besides the clear effect of age.

## Conclusion

We herein reported data on the largest cohort of CVID patients ever investigated for CKD, finding a prevalence (6.3%) higher than that reported in previous studies involving smaller cohorts. The increasing prevalence of CKD observed over time in CVID, as well as in the normative population, may reflect an undetected and consequently untreated disease, but at the same time raises the attention on ageing and age-related issues in a cohort of patients whose survival is increasing, thanks to the impact of anti-infectious and immunomodulatory treatments. Apart from aging, our data also suggest that renal function needs to be evaluated in all CVID patients, and particularly in those prone to a higher “inflammatory” burden. Further studies are warranted to confirm these results and in particular, the role of neutrophil-to-lymphocyte ratio in the prediction of renal damage.

## Electronic Supplementary Material

Below is the link to the electronic supplementary material.

Supplementary Material 1

## Data Availability

No datasets were generated or analysed during the current study.

## Abbreviations

CVID: Common variable immunodeficiency
PADs: Primary Antibody Deficiencies
CKD: Chronic kidney disease
IVIG: Intravenous immunoglobulin
SCIG: Subcutaneous immunoglobulin
AKI: Acute kidney injury
BMI: Body mass index
eGFR: Estimated glomerular filtration rate
IBD: Inflammatory bowel disease
UTI: Urinary tract infection
Hb: Hemoglobin
Na: Sodium
K: Potassium
Ca: Calcium
P: Phosphorus
IgRT: IgG replacement therapy
ILD: Interstitial lung disease
COPD: Chronic obstructive pulmonary disease
ALC: Absolute lymphocytes count
AI: Autoimmune cytopenia
