# Supplementary material for: Chronic Kidney Disease in Common Variable Immunodeficiency: a Multicenter Study
Source: J Clin Immunol. 2025 May 23;45(1):97. doi: 10.1007/s10875-025-01890-2 (PMC12102111; doi:10.1007/s10875-025-01890-2)
Supplement: Supplementary file 1 — Supplementary Material 1 [file 10875_2025_1890_MOESM1_ESM.pdf]

SUPPLEMENTARY DATA

Supplementary Methods

To estimate GFR, we used the new Chronic Kidney Disease Epidemiology Collaboration equation expressed as a single equation, using the Scr value as follows: eGFRcr=142 × min(Scr/κ, 1)α × max(Scr/κ,1) − 1.200×0.9938Age×1.012 [if female]; where Scr is standardized serum creatinine in mg/dL, κ is 0.7 for females or 0.9 for males, α is − 0.241 for females or − 0.302 for males, min(Scr/κ, 1) is the minimum of Scr/κ or 1.0, max(Scr/κ, 1) is the maximum of Scr/κ or 1.0 [23]

Supplementary Tables Legends:

Table S1: Comparison of laboratory urine test in the total cohort and between CVID patients with and without CKD.

Table S2: Comparison of laboratory data and B cells subpopulations between CVID patients with infection-only and complicated phenotype.

Table S3: Comparison of demographic characteristics, clinical, laboratory, instrumental variables and treatments between CVID patients with or without CKD, adjusted for age.

Table S4: Multivariate analysis: demographic, laboratory, clinical and therapeutic variables associated with CKD after correction for age.

Table S1

Urine test	Total cohort (n=367)	No CKD (n=344)	CKD (n=23)	p value	OR (IC95%)
Ph, median (IQR)	5.5 (5.5 - 6)	5.5 (5.5-6.2)	5.5 (5.25-5.5)	0.054	0.36 (0.13-1.02)
Urinary specific weight, median (IQR)	1019 (1013 - 1024)	1019 (1014-1025)	1015 (1011-1020)	0.685	0.99 (0.93-1.05)
Urinary Sediment, n (%)	76 (20.7)	72	4	1.000	0.874 (0.27-2.83)
Erythrocyte, cells/microL, median (IQR)	1 (0 - 8.5)	1 (0-9)	1 (0-4.5)	0.358	0.96 (0.89-1.04)
Leukocytes cells/microL, median (IQR)	2 (0 - 10)	2 (0-11)	3 (1.5-6)	0.778	0.99 (0.99-1.00)
Spot proteinuria, n (%)	28 (7.6)	28 (8.1)	0 (0)	0.382	0.246 (0.01-4.23)
Glicosuria, n (%)	3 (0.8)	3 (0.9)	0 (0)	1.000	2.39 (0.12-48.5)
Nitrites, n (%)	8 (2.2)	7 (2)	1 (4.3)	0.364	2.60 (0.30-22.8)
Bacterial flora, n (%)	34 (9.3)	32 (9.3)	2 (8.7)	1.000	1.10 (0.24-5.16)

Urinary sediment, spot proteinuria, glycosuria, nitrites and bacterial flora are reported as qualitative variables, considering either their presence or absence. We defined urinary sediment as the presence of spot proteinuria and/or urinary erythrocytes concentration above 10 mg/dl; proteinuria as the presence of urinary protein concentration above 20 mg/dl, glycosuria as the presence of urinary glucose concentration above 10 mg/dl and bacterial flora as the presence of bacterial cells above 1000 cells/microL.

Table S2

	**Infection-only phenotype (n=189)**	**Complicated phenotype (n=178)**	**p value**
Age, median (IQR)	53 (39-61.5)	51 (38-63)	0.407
CKD, n (%)	9 (4.8)	14 (7.9)	0.225
Creatinine (mg/dl), median (IQR)	0.77 (0.69-0.90)	0.79 (0.68-0.94)	0.715
eGFR (mL/min/1.73m2), median (IQR)	99 (84-108)	96 (84-110)	0.852
Hb (g/dl), median (IQR)	**14 (13.1-15.1)**	**13.4 (12.2-14.6)**	**<0.001**
Platelet-to-lymphocytes ratio, median (IQR)	121 (96-156)	114 (80-172)	0.189
Neutrophil-to-lymphocyte ratio, median (IQR)	2 (1.5-3)	2.2 (1.5-3.2)	0.513
ALC (cells/mm^3), median (IQR)	**1670 (1276-2100)**	**1368 (880-1900)**	**<0.01**
T cells (%), median (IQR)	**76 (70-80)**	**79 (70-85)**	**0.018**
B cells (%), median (IQR)	**10 (6-14)**	**8 (3-12)**	**0.010**
Sm B cells (%), median (IQR)	**3 (1-6)**	**1.6 (0.7-4)**	**0.012**
Mz B cells (%), median (IQR)	**10 (4-21)**	**5 (1.4-15)**	**0.006**
CD21low B cells (%), median (IQR)	5.7 (2.6-10.6)	6.6 (3-14)	0.212
Naive B Cells (%), median (IQR)	58 (31-78)	70 (40-87)	0.104
Tr B cells (%), median (IQR)	1 (0.3-2.3)	0.9 (0.2-2.6)	0.833

B cells sub-populations were identified according to EUROclass study: Sm B cells: Switched memory IgD−IgM−CD27+; CD21low B cells: Activated CD21lowCD38low; MZ B cell: Marginal zone IgD+IgM+CD27+; Tr B cells: Transitional CD38++IgMhigh; Naive B cells: Naïve IgD+IgM+CD27− [22]. Statistically significant results in bold.

Table S3

	p value	OR (IC95%)
Female sex	0.522	0.74 (0.29-1.88)
Hypertension	0.357	1.55 (0.61-3.96)
Diabetes	0.126	2.56 (0.77-8.54)
Dyslipidemia	0.224	1.79 (0.70-4.56)
Complicated phenotype	0.103	2.17 (0.86-5.48)
Splenomegaly	0.154	1.99 (0.77-5.13)
AI cytopenia	**0.008**	**3.54 (1.38-9.08)**
Enteropathy	0.050	0.22 (0.05-1.00)
ILD	0.192	2.01 (0.70-5.75)
Bronchiectasis	0.960	0.98 (0.39-2.45)
COPD	0.170	1.94 (0.75-4.98)
AI systemic disease	0.422	1.94 (0.38-9.74)
Recurrent UTI	0.792	0.84 (0.22-3.15)
Antibiotic prophylaxis	0.145	2.11 (0.77-5.76)
Trimethoprim-sulfamethoxazole	**0.039**	**6.51 (1.1-38.7)**
Azithromycin	0.422	0.56 (0.13-2.31)
IVIG	0.052	2.47 (0.99-6.13)
maltose-stabilized product	0.644	0.73 (0.19-2.76)
SCIG	0.760	0.86 (0.34-2.19)
Median CVID duration	0.961	1.00 (0.96-1.03)
Median IgG at diagnosis	0.234	0.99 (0.99-1.00)
Median IgA at diagnosis	0.436	1.01 (0.99-1.02)
Median IgM at diagnosis	0.619	1.00 (0.99-1.00)
Median IgG TL	0.825	1.00 (0.99-1.00)

Median IgRT mg/kg/month	0.662	1.00 (0.99-1.00)
Changes in cortico-medullary differentiation and longitudinal bipolar diameters at Ultrasound	**0.003**	**18.34 (2.65-127)**
Any other US abnormalities*	0.217	1.80 (0.71-4.55)
ALC median [IQR]	0.302	1.00 (0.99-1.00)
Median T cells %	0.429	1.01 (0.98-1.03)
Median CD4+T cells %	0.855	0.99 (0.94-1.05)
Median CD8+T cells %	0.178	1.03 (0.99-1.08)
Median B cells %	**0.017**	**0.85 (0.75-0.97)**
Median Sm B cells %	0.491	0.93 (0.76-1.14)
Median CD21low B cells %	0.916	1.00 (0.95-1.05)
Median Mz B cells %	0.547	0.98 (0.93-1.03)
Median Tr B cells %	0.153	5.08^{-4} (1.55^{-8}-16.65)
Median Naive B cells %	0.780	1.00 (0.98-1.03)
Median neutrophil-to-lymphocyte ratio	**0.010**	**1.17 (1.04-1.32)**
Median platelet-to-lymphocytes ratio	0.704	1.00 (0.99-1.00)

*mild pyelectasis, nephrolithiasis, cysts.
B cells sub-populations were identified according to EUROclass study: Sm B cells: Switched memory IgD−IgM−CD27+; CD21low B cells: Activated CD21lowCD38low; MZ B cell: Marginal zone IgD+IgM+CD27+; Tr B cells: Transitional CD38++IgMhigh; Naive B cells: Naïve IgD+IgM+CD27− [22].
Statistically significant results in bold.

Table S4

	p value	OR [IC95%]
Age	0.089	1.055 [0.99-1.13]
Neutrophil-to-lymphocytes ratio	0.188	1.341 [0.87-2.07]
B cell %	0.263	0.890 [0.73-1.09]
AI cytopenia	**0.024**	**21.391 [1.41-136.80]**
Trimethoprim-sulfamethoxazole antibiotic prophylaxis	0.771	1.441 [0.12-16.96]

Statistically significant results in bold. AUC 0.889
